# Identification and Functional Analysis of a Novel Cytochrome P450 Gene *CYP9A105* Associated with Pyrethroid Detoxification in *Spodoptera exigua* Hübner

**DOI:** 10.3390/ijms19030737

**Published:** 2018-03-05

**Authors:** Rui-Long Wang, Shi-Wei Liu, Scott R. Baerson, Zhong Qin, Zhi-Hui Ma, Yi-Juan Su, Jia-En Zhang

**Affiliations:** 1 Guangdong Engineering Research Center for Modern Eco-Agruculture and Circular Agriculture, Guangzhou 510642, China; rlw2009@scau.edu.cn (R.-L.W.); 13711379414@163.com (S.-W.L.); q_breeze@scau.edu.cn (Z.Q.); 13924082629@163.com (Z.-H.M.); syj@scau.edu.cn (Y.-J.S.); 2 Key Laboratory of Agro-Environment in the Tropics, Ministry of Agriculture, South China Agricultural University, Guangzhou 510642, China; 3 United States Department of Agriculture-Agricultural Research Service, Natural Products Utilization Research Unit, University of Mississippi, Oxford, MS 38677, USA; scott.baerson@ars.usda.gov; 4 Key Laboratory of Agroecology and Rural Environment of Guangdong Regular Higher Education Institutions, South China Agricultural University, Guangzhou 510642, China

**Keywords:** *Spodoptera exigua*, cytochrome P450, pyrethroids, RNA interference, CYP9A105

## Abstract

In insects, cytochrome P450 monooxygenases (P450s or CYPs) are known to be involved in the detoxification and metabolism of insecticides, leading to increased resistance in insect populations. *Spodoptera exigua* is a serious polyphagous insect pest worldwide and has developed resistance to various insecticides. In this study, a novel CYP3 clan P450 gene *CYP9A105* was identified and characterized from *S. exigua*. The cDNAs of *CYP9A105* encoded 530 amino acid proteins, respectively. Quantitative real-time PCR analyses showed that *CYP9A105* was expressed at all developmental stages, with maximal expression observed in fifth instar stage larvae, and in dissected fifth instar larvae the highest transcript levels were found in midguts and fat bodies. The expression of *CYP9A105* in midguts was upregulated by treatments with the insecticides *α*-cypermethrin, deltamethrin and fenvalerate at both LC_15_ concentrations (0.10, 0.20 and 5.0 mg/L, respectively) and LC_50_ concentrations (0.25, 0.40 and 10.00 mg/L, respectively). RNA interference (RNAi) mediated silencing of *CYP9A105* led to increased mortalities of insecticide-treated 4th instar *S. exigua* larvae. Our results suggest that *CYP9A105* might play an important role in *α*-cypermethrin, deltamethrin and fenvalerate detoxification in *S. exigua*.

## 1. Introduction

Cytochrome P450 monooxygenases (P450s or CYPs) represent an important supergene family of detoxification enzymes involved in the metabolism of a wide range of endogenous and exogenous compounds, including plant allelochemicals and insecticides [1,2,3,4,5,6]. In insects, P450 genes can be assigned to one of four clades: CYP2, CYP3, CYP4 and the mitochondrial CYP clade [1,2]. The CYP3 clan is comprised of a large number of insect CYPs and is subdivided into the CYP6, CYP9, CYP28, CYP308–310, CYP317, CYP321, CYP324, CYP329, CYP332, CYP336–338 and CYP345–348 families [5]. Several CYP9 family members are known to participate in detoxification pathways associated with insecticide resistance [7,8,9,10,11,12]. *CYP9A1* in *Heliothis virescens* was the first member of the CYP9 family shown to be transcriptionally induced in response to thiodicarb exposure, suggesting a potential role for CYP9A1 in insecticide metabolism [9].

The beet armyworm, *Spodoptera exigua* (Hübner) (Lepidoptera: Noctuidae) is a highly polyphagous pest which infests more than 138 host species representing 35 different plant families worldwide [13]. *S. exigua* is responsible for considerable economic losses to important crops such as corn, cotton, peanut, sorghum, soybean and tobacco [14]. Excessive and frequent insecticide applications have caused *S. exigua* to develop high resistance levels to pyrethroid insecticides in many field populations [15,16,17]. At present, deltamethrin, α-cypermethrin and fenvalerate are widely used pyrethroids insecticide in agriculture to effectively control *S. exigua* [15,16,17]. Insecticide resistance in *S. exigua* has been documented throughout the world, including the United States, China, Taiwan, Guatemala, Mexico, Thailand, Spain and Pakistan [15,18]. For pyrethroids, compared with a susceptible Lab-Pk population, the resistance ratios of the field populations of *S. exigua* from four districts of the Punjab, Pakistan were in the range of 7–105-fold for deltamethrin, 12–136-fold for cypermethrin [15]. Meanwhile, *S. exigua* became highly resistant to *β*-cypermethrin in 2008 with resistance ratios ranging from 95.31-, 437.97-, 98.18-fold for the Taian, Zhangqiu, Anqiu populations [18]. The resistance of *S. exigua* to *β*-cypermethrin in Zhangqiu rapidly increased from 437.97-fold in 2008 to 1304.40-fold in 2009, and to 1095.31-fold in 2010 [18]. A study indicated that P450s was partially responsible for a very high level of resistance to pyrethroids in *S. exigua* from Mexico [16]. Synergism studies also indicate that metabolic detoxification by P450s may be involved in resistance to pyrethroids in the Pakistani populations of *S. exigua* [15,16].

In this study, we examined the potential roles played by a cytochrome P450 gene in conferring detoxification to pyrethroid insecticides in *S. exigua*. RNA-Seq analysis was first used to identify P450 sequences expressed in fourth instar larval midgut tissues. A novel P450 gene belonging to CYP9 family and designated *CYP9A105* was subsequently isolated and characterized. Quantitative real-time RT-PCR (qRT-PCR) studies were performed to investigate the organ- and developmental stage-specific expression patterns of *CYP9A105* and its potential roles in the detoxification of three synthetic pyrethroid insecticides were tested using RNAi, followed by bioassays. These results provide significant insight into the function of *S. exigua CYP9A105* in the deltamethrin, α-cypermethrin and fenvalerate detoxification.

## 2. Results

### 2.1. Identification and Characterization of CYP9A105

**Figure 1:**
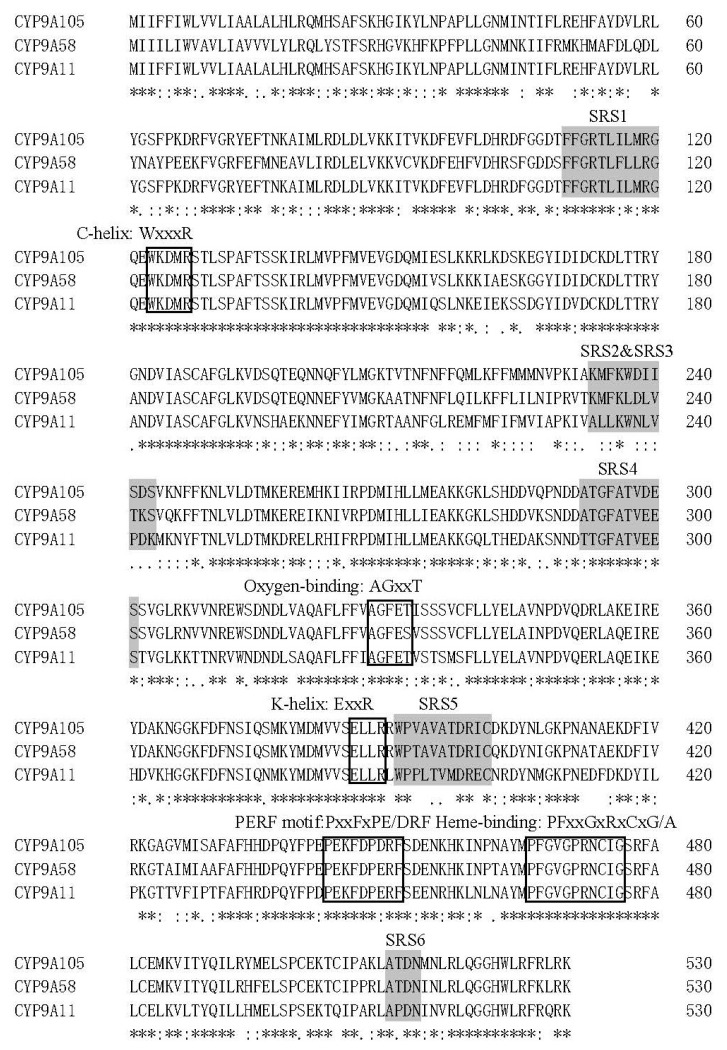
Comparison of the deduced amino acid sequences of *Spodoptera exigua CYP9A105*, *Spodoptera littoralis CYP9A51* (GenBank accession number JX310084.1) and *Spodoptera frugiperda CYP9A58* (GenBank accession number KJ671577.1). Substrate recognition sites (SRS) are indicated with gray boxes. Identical amino acid residues are marked with “*”, and conserved residues with “:”. The conserved P450 Helix C (WxxxR), Oxygen-binding motif (AGxxT), Helix K (ExxR), PERF motif (PxxFxPE/DRF) and heme-binding domain (PFxxGxRxCxG/A) are also shown.

To identify cytochrome P450 genes with potential roles in the detoxification of pyrethroid insecticides in *S. exigua*, RNA-Seq analysis was first performed on fourth instar larval midgut samples. The resulting transcriptome data was mined by BLAST analyses for P450-like sequences, and 60 unique sequences were retrieved, one of which were identified as putative *CYP9A* subfamily members. The full open reading frames (ORFs) for these CYP9A-like sequences were then confirmed by RT-PCR and sequence analysis and designated as *CYP9A105* (GenBank accession number KY348418.1). The length of nucleotide sequences is 1581 and the molecular weight of the predicted proteins is 61.434 kDa. The ORF encodes a predicted protein of 530 amino acids. The estimated isoelectric point (pI) value is 8.84. Signal peptides with 17 amino acids were found at the N-terminal ends of *CYP9A105*. Sequence alignment showed that *CYP9A105* from *S. exigua* shared 82.5% identity with *CYP9A51* of *Spodoptera littoralis*, 79.6% identity with *CYP9A58* of *Spodoptera frugiperda* and 78.3% identity with *CYP9A11* of *S. exigua.* The predicted amino acid sequences contained the conserved cysteine residue required for heme binding, as well as the highly conserved hydrogen bonding regions, including WKDMR (WXXXR) in Helix-C, AGFET (AGxxT) in the Helix-I, ELLR (ExxR) in Helix-K. The Meander motif PEKFDPDRF (PxxFxPxxF) and the P450 signature motif FGVGPRNCIG (FxxGxRxxxG) were also identified (Figure 1) [8,11].

### 2.2. Tissue-Specific and Development Expression of CYP9A105

**Figure 2:**
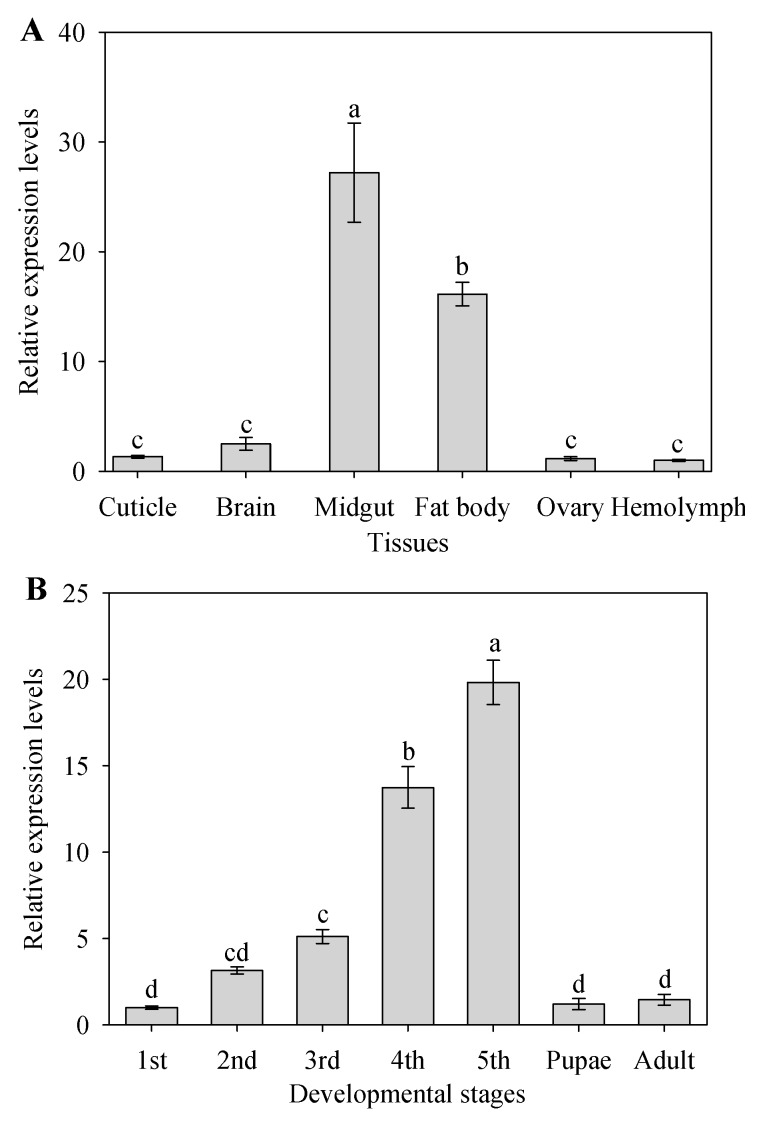
Expression of *CYP9A105* in different tissues (**A**) and different developmental stages (**B**) of *Spodoptera exigua*. Real-time RT-qPCR analysis was used to determine the relative transcript levels for each gene. Data shown represent means ± SE derived from three biological replicates. Different letters above bars indicate significant differences (*p* < 0.05) according to the Duncan’s multiple range test.

The steady-state transcript levels of *CYP9A105* were examined in six different tissues using RT-qPCR. The analysis showed that the *CYP9A105* was predominantly expressed in fat body and midgut, and also detectable in cuticle, brain, ovary and hemolymph at significantly lower levels. The higher expression levels of *CYP9A105* were observed in midgut and fat bodies were approximately 27.2- and 16.1-fold more than in the hemolymph, respectively (Figure 2A).

Further RT-qPCR analysis of *CYP9A105* expression levels indicated that its expression varied significantly throughout the seven life stages of *S. exigua* (Figure 2B). Maximal expression of *CYP9A105* was detected at the fifth instar larvae stage (19.8-fold more than the first instar larvae stage), followed by fourth instar larvae (13.7-fold more than in the first-instar larvae) and third instar larvae (5.1-fold more than in the first-instar larvae) (Figure 2B).

### 2.3. Effect of Insecticide Exposure on Expression Level CYP9A105

**Figure 3:**
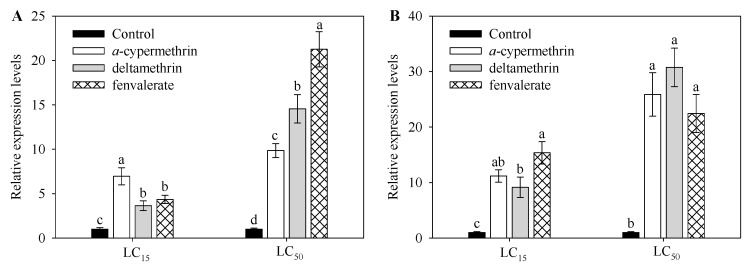
Effects of α-cypermethrin, deltamethrin or fenvalerate on *CYP9A105* expression in midguts (**A**) and fat body (**B**) *Spodoptera exigua*. Insects were treated with insecticides at LC_15_ doses (α-cypermethrin, deltamethrin and fenvalerate at 0.10, 0.20 and 5.00 mg/L, respectively) and LC_50_ doses (*α*-cypermethrin, deltamethrin and fenvalerate at 0.25, 0.40 and 10.00 mg/L, respectively), for comparison with control treatments. RT-qPCR analysis was used to determine relative transcript levels. Data shown are mean ± SE derived from three biological replicates. Different letters above bars indicate significant differences (*p* < 0.05) according to the Duncan’s multiple range test.

Three commonly used insecticides—deltamethrin, α-cypermethrin or fenvalerate—were selected to examine their effects on the expression of *CYP9A105* in midguts of *S. exigua* (Figure 3)*.* When *S. exigua* was exposed to α-cypermethrin, deltamethrin or fenvalerate at the LC_15_ concentration, expression of *CYP9A105* increased by 7.0-, 3.6- and 4.3-fold (Figure 3A) in the midguts. α-cypermethrin, deltamethrin, or fenvalerate significantly increased *CYP9A105* expression levels at the LC_50_ dose relative to control treatments. The maximum increase observed for fenvalerate was 21.3-fold in the midguts (Figure 3A). α-cypermethrin, deltamethrin, or fenvalerate significantly increased *CYP9A105* in the fat body of *S. exigua* at LC_15_ and LC_50_ concentration (Figure 3B). The maximum increase observed for deltamethrin was 30.8-fold in the fat body at the LC_50_ concentration (Figure 3B).

### 2.4. Effect of Piperonyl Butoxide on the Toxicity of Pyrethroids

Synergism bioassays were performed to determine the effects of the P450 inhibitor piperonyl butoxide (PBO) on the toxicity of α-cypermethrin, deltamethrin and fenvalerate to fourth instar *S. exigua* larvae. The LC_50_ of α-cypermethrin, deltamethrin and fenvalerate were 0.27, 0.43 and 10.92 mg/L when used alone, respectively. The addition of PBO reduced the LC_50_ of α-cypermethrin, deltamethrin and fenvalerate to 0.06, 0.11 and 2.12 mg/L, respectively. The synergist ratios were 4.63-, 3.78- and 5.15-fold at the LC_50_ level, respectively (Table 1). These results indicated that treatment with PBO to *S. exigua* has significantly increased the toxicity of α-cypermethrin, deltamethrin and fenvalerate.

### 2.5. Functional Analysis of CYP9A105 by RNAi

**Figure 4:**
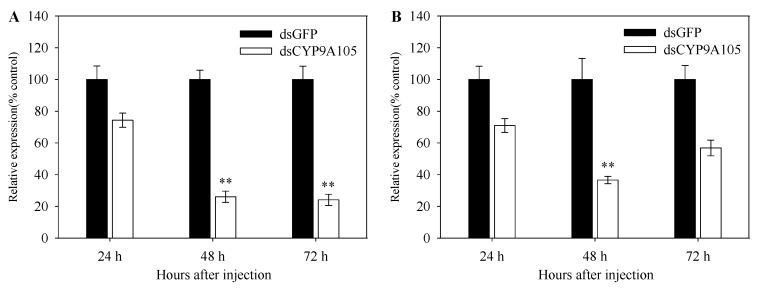
*Spodoptera exigua CYP9A105* relative transcript levels in midguts (**A**) and fat bodies (**B**) following injection of dsGFP or dsCYP9A105. Data shown are mean ± SE derived from three biological replicates. Asterisk indicates a significant difference between relative transcript levels of dsGFP injected individuals compared with dsCYP9A105 injected individuals (*p* < 0.05, Student’s *t*-test, ** *p* < 0.01).

To determine whether reduced expression of *CYP9A105* has an effect on the susceptibility of *S. exigua* to pyrethroids, RNAi-mediated silencing of this gene was performed on *S. exigua* fourth instar larvae exposed to α-cypermethrin, deltamethrin, or fenvalerate. After dsRNA injection, *CYP9A105* expression levels were reduced by 74.0% and 75.9% in the midgut of *S. exigua* at 48 and 72 h, respectively (Figure 4A). However, expression level of *CYP9A105* in the fat body of *S. exigua* was decreased to 36.6% after injection with dsCYP9A105 for 48 h (Figure 4B). This result demonstrated that RNAi effectively suppressed the expression of *CYP9A105* in *S. exigua* under the conditions employed.

**Figure 5:**
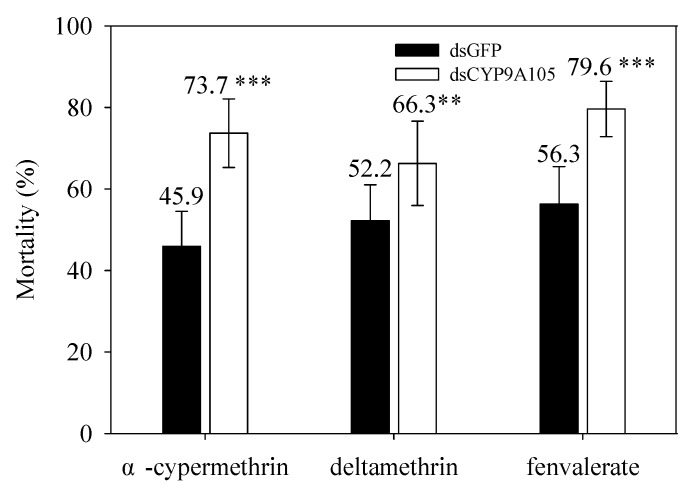
Effect of dsCYP9A105 injection on susceptibility of fourth-instar larvae of *S. exigua* to *α*-cypermethrin, deltamethrin and fenvalerate. Following injection with dsCYP9A105 or double-stranded green fluorescent protein (dsGFP), larvae were maintained on the different insecticide-treated cabbage leaves for 48 h. Data shown are mean ± SE derived from three biological replicates. Asterisk indicates a significant difference between dsGFP injected individuals compared with dsCYP9A105 injected individuals (*p* < 0.05, Student’s *t*-test, ** *p* < 0.01, *** *p* < 0.001).

Mortality of fifth-instar larvae of *S. exigua* (without exposure to any insecticide) injected with dsGFP or dsCYP9A105 and then exposed to either α-cypermethrin, deltamethrin, or fenvalerate are shown in Figure 5. This result indicated that delivery of dsCYP9A105 to larvae significantly enhanced the insecticidal activity of all three pyrethroids (Figure 5). Following exposure to *α*-cypermethrin (0.25 mg/L), deltamethrin (0.40 mg/L), or fenvalerate (10.00 mg/L) at the LC_50_ doses, larvae injected with dsCYP9A105 (compared to dsGFP) significantly increased larval mortality caused by *α*-cypermethrin (from 45.9% to 73.7%), deltamethrin (from 52.2% to 66.3%) or fenvalerate (from 56.3% to 79.6%). These results revealed that injection with dsCYP9A105 combined with exposure to either deltamethrin, *α*-cypermethrin or fenvalerate resulted in higher mortality than was observed for larvae exposed to these insecticides in the absence of dsCYP9A105 injections. Taken together, our results strongly suggest that *CYP9A105* might play a role in the detoxification of pyrethroid insecticides in *S. exigua*.

## 3. Discussion

Herbivorous insects have evolved a variety of mechanisms to adapt to insecticides; foremost among these include increased activities of detoxifying enzymes and decreased sensitivities of insecticide target sites [5,7,19,20]. Among the elucidated metabolic mechanisms of resistance known to occur for pyrethroids, the most common involve enhanced detoxification by inducible carboxylesterases, glutathione S-transferases and cytochrome P450s [19,20]. The insect CYP9 family is well known to play an important role in insecticide resistance, and some members have been shown to be inducible by pesticides [3,5,10]. *S. exigua* has developed resistance against most of the insecticides (e.g., organophosphorus, organochlorine and pyrethroids) commonly used for its control [15,17,18,21]. Furthermore, the insecticides used in this study—deltamethrin, *α*-cypermethrin and fenvalerate—are three pyrethroid insecticides which have been widely used for controlling *S. exigua* in vegetable plantations [15,17,18]. To further understand the relationship between this P450 subfamily and insecticide resistance in *S. exigua*, a novel CYP9A member, *CYP9A105,* was identified and characterized. Remarkably, the SRS4 and SRS5 sequences in *CYP9A105* of *S. exigua* (Figure 1) are very similar to those of *CYP9A51* of *S. littoralis* and *CYP9A58* of *S. frugiperda*. The expression of *CYP9A39* in midguts of *S. litura* has been shown to be increased in populations fed on diets supplemented with lead for several generations, and these populations also showed enhanced tolerance to the insecticide cypermethrin [22]. CYP9A105 of *S. exigua* shares 79.6% amino acid identity with CYP9A58 of *S. frugiperda*, which has been shown to be induced by both methoxyfenozide and deltamethrin [23,24]. Based on these similarities as well as the RNAi bioassay results obtained in the present work, we suggest that *CYP9A105* possesses similar substrate preferences for insecticides. This suggestion is also supported by the observation that transcript levels of *CYP9A105* in *S. exigua* larvae are similarly up-regulated in midguts in response to all three insecticides (Figure 3).

P450 genes are known to be expressed throughout insect development and are distributed in virtually all tissue types, and their tissue- and developmental stage-specific expression patterns can often provide clues concerning their physiological roles [5,8,19]. For example, various studies have shown that midguts and fat bodies of insect larvae play central roles in xenobiotic metabolism, thus detoxifying P450 enzymes may be highly expressed in these organs [5,19]. We therefore characterized the expression profiles of *CYP9A105* in *S. exigua*, and importantly, these results revealed that maximal expression for *CYP9A105* occurred in fifth instar stage larval midguts and fat bodies (Figure 2). Similar expression patterns have been reported for *CYP9A40* from *S. litura* [3], *CYP9A61* from *Cydia pomonella* [11], multiple *S. frugiperda* CYP9 family members (*CYP9A24*, *CYP9A26*, *CYP9A31*, *CYP9A52*, *CYP9A51*) and *S. exigua CYP9A9* [23,24,25]. The fact that *S. exigua CYP9A105* is primarily expressed in these tissues would be consistent with the hypothesis that *CYP9A105* could play a role in the detoxification of xenobiotic compounds, such as insecticides encountered during feeding.

RT-qPCR results obtained in this work also demonstrated that the expression levels of *CYP9A105* were significantly increased in *S. exigua* larval midguts following exposure to deltamethrin, *α*-cypermethrin and fenvalerate (Figure 3). A large number of studies have reported increased expression of CYP9 family genes in larvae following insecticide exposure. For example, *CYP9A40* of *S. litura* has been shown to be induced by deltamethrin and methoxyfenozide, and functional studies also indicate that this enzyme participates in the detoxification of these compounds [4]. In *Apis mellifera*, *CYP9Q3* is induced by tau-fluvalinate, and also likely contributes to tau-fluvalinate tolerance by participating in its metabolism [12]. *CYP9A12* has been shown to be involved in pyrethroid resistance in *Bombyx mori* [26], and in *Helicoverpa armigera*, *CYP9A12* has been shown to be induced by the pyrethroid deltamethrin in the mid guts [27]. These and other examples from the literature have demonstrated that in insects, P450s can frequently be induced by insecticides, and may also participate in the metabolism of the compounds eliciting their induction. 

RNAi is a universal gene-silencing technology and has been successfully used to investigate the function of P450s in many insect species [3,8,20,28,29,30]. For example, the mortality of *Locusta migratoria* exposed to fluvalinate was shown to increase from 29.8% to 53.0% after *CYP9AQ1* was silenced using RNAi, and exposure to permethrin and deltamethrin increased mortality from 27.7% to 58.3% and 27.7% to 77.7%, respectively, after *CYP9A3* was silenced using RNAi, thus indicating a role for *CYP9AQ1* and *CYP9A3* in pyrethroid insecticide detoxification [10]. Additionally, simultaneous silencing of six different P450 genes in a fenpropathrin-resistant strain of *Tetranychus cinnabarinus* (*CYP389B1*, *CYP392A26*, *CYP391A1*, *CYP384A1*, *CYP392D11* and *CYP392A28*) by RNAi had an even greater effect on fenpropathrin resistance than silencing them individually, indicating that these six P450 genes collaboratively participate in conferring fenpropathrin resistance to this organism [20]. In the current work, RNAi-mediated silencing of *CYP9A105* was shown to significantly increase the mortality of *S. exigua* larvae exposed to deltamethrin, *α*-cypermethrin and fenvalerate, strongly suggesting that *CYP9A105* may play an important role in their metabolic detoxification, and assist in conferring resistance against these insecticides.

At present, the rapid development of resistance in many insect and mite species to various insecticides poses a significant threat to agriculture worldwide [6]. *S. exigua* represents one of the most agriculturally important phytophagous insect pests which feed on a wide range of crops, and excessive insecticide use has led to the development of resistance against most of the insecticides [15,17,18,21]. Furthermore, the insecticides used in this study including deltamethrin, *α*-cypermethrin and fenvalerate, are three pyrethroid insecticides which have been widely used for controlling *S. exigua* in vegetable plantations [15,17,18]. P450 detoxification systems undoubtedly play a central role in the adaptation of *S. exigua* to these and other agricultural insecticides, and the findings of the present study provide new insights concerning the critical roles likely played by CYP9A105 in this process.

## 4. Materials and Methods

### 4.1. Insects

Eggs of *S. exigua* were provided by the Insectarium of the Institute of Entomology, Sun Yat-sen University (Guangzhou, China). The eggs were incubated at 25 ± 2 °C with a 14:10-h (light:dark) photoperiod and 70 ± 5% humidity in an insectary without exposure to insecticides. After hatching, *S. exigua* were reared with an artificial diet [31] under the same conditions.

### 4.2. RNA-Seq Library Preparation

Total RNA was prepared from *S. exigua* fourth instar midguts using the Trizol reagent according to the manufacturer’s protocol (Invitrogen, Burlington, ON, Canada), and treated with DNase I (Qiagen, Valencia, CA, USA). The yield and purity of RNA was determined by UV-Vis spectroscopy, and RNA integrity was examined by agarose gel electrophoresis. 3 μg of total RNA was used for polyA + RNA isolation to prepare a nondirectional Illumina RNA-Seq library using an Illumina TruSeq™ RNA Sample Preparation Kit (Illumina, San Diego, CA, USA) following the manufacturer’s recommendations. The library was quantified and then sequenced using an Illumina HiSeq™ 2000 sequencer. The 29,716,661 reads obtained generated 31,943 unigenes following de novo transcriptome assembly. Using the BLASTX algorithm, all unigenes were compared against the Nr, Swiss-Prot, GO, COGs, KOG and KEGG protein databases. Among the 31,943 unigenes generated from the *S. exigua* midgut library, a novel CYP9 family P450 gene (*CYP9A105*) were identified.

### 4.3. Cloning of CYP9A105

Total RNAs used for RT-qPCR analyses were extracted using TRIzol reagent (Invitrogen, Carlsbad, CA, USA), and the yield and purity of RNAs were determined by UV-Vis spectroscopy, and RNA integrity was examined by agarose gel electrophoresis. Purified RNAs were then treated with DNase I (Invitrogen) and reverse-transcribed using a Thermo Script RT-PCR system (Life Technologies, Grand Island, NY, USA).

A CYP9A subfamily member-like sequences were identified by BLASTN and TBLASTN analyses (http://blast.ncbi.nlm.nih.gov) using the sequences derived from the fourth instar midgut RNA-seq analysis. To confirm the predicted coding sequences of *CYP9A105*, specific primers (CYP9A105FullF and CYP9A105FullR) (Table 2) designed from start codon to stop codon were used to amplify the full-length cDNAs by reverse transcription-PCR (RT-PCR), using cDNA templates prepared from *S. exigua* fourth instar midguts. The following thermal cycling parameters were used: 95 °C for 1 min, 35 cycles of 95 °C for 30 s, 60 °C for 90 s and 72 °C for 60 s, with a final extension step of 72 °C for 10 min. The PCR products were examined by electrophoresis on 1.2% agarose gels, then subcloned using the pGEM-T Easy Vector (Promega, Madison, WI, USA) and sequenced using an ABI 377 DNA sequencer (Perkin Elmer, Foster City, CA, USA).

### 4.4. Bioinformatics

The full-length cDNA of *CYP9A105* deduced amino acid sequence were determined with Expert Protein Analysis System (http://www.expasy.org/tools/pi_tool.html). Deduced amino acid sequences of *CYP9A105* from *S. exigua* and other insect species were aligned by using ClustalX [32]. Signal peptides were predicted using the SignalP 3.0 Server (http://www.cbs.dtu.dk/services/SignalP-3.0/). The molecular weights and isoelectric points (pI) were calculated using the ExPASy Protemics Server (http://cn.expasy.org/tools/pi_tool.html).

### 4.5. Determining Tissue- and Developmental Stage-Specific Expression Pattern of CYP9A105

To determine tissue-specific expression patterns for the *CYP9A* sequences, six tissues (cuticle, brain, midgut, fat body, ovary and hemolymph) were dissected at day 2 from fifth instar *S. exigua* larva. For developmental stage-specific expression analyses, samples were collected at day 2 of each developmental stage, including first to fifth instar larvae, pupae, and adults of *S. exigua*. Three biological replicates were collected for all samples. RNA isolation and cDNA synthesis were performed as described above.

The relative expression levels of *CYP9A105* were quantified by real time quantitative reverse transcription (RT-qPCR), using gene specific PCR primers (CYP9A105F1 and CYP9A105R1) (Table 2). RT-qPCR was performed using an MJ Research Opticon instrument (Bio-Rad, Inc., Hercules, CA, USA) with SYBR Green I Master Mix (Roche Diagnostics Corp., Indianapolis, IN, USA). RT-qPCR reaction mixtures contained 10.0 μL of 2× SYBR Green I master mix (Roche), 1 μL of cDNA template, 10 pmoles of each primer, and sufficient RNAse-free water to obtain a final volume of 20 μL. The *β-actin* (GenBank accession AY507963) gene [33] was used as the internal standard (Table 2). The thermal cycling parameters used were: 94 °C for 3 min, followed by 40 cycles of 94 °C for 30 s, 60 °C for 30 s and 72 °C for 30 s. To assess the specificity of the RT-qPCR amplifications, a melt curve analysis was performed at the end of the runs. All assays were performed in triplicate using three independent biological replicate samples. The relative expression values were calculated using the 2^−^^∆∆*C*t^ method as previously described [34].

### 4.6. Insecticide Exposures

Piperonyl butoxide (PBO, 90%) was obtained from Sigma-Aldrich (St. Louis, MO, USA). Stock solutions for the three insecticides used, deltamethrin (Jiangsu Yangnong Chemical Group Co., Ltd., Yangzhou, China), *α*-cypermethrin (Nanjing Ronch Chemical Co., Ltd., Nanjing, China) and fenvalerate (Jiangsu Lanfeng Bio-chemical Co., Ltd., Xuzhou, China) were prepared by dissolving all active ingredients in acetone to a final concentration of 10.00 mg/L. The doses used for lethal concentration-15% (LC_15_) treatments were 0.10, 0.20 and 5.00 mg/L for *α*-cypermethrin, deltamethrin and fenvalerate, respectively, and LC_50_ doses used were 0.25, 0.40 and 10.00 mg/L for *α*-cypermethrin, deltamethrin and fenvalerate, respectively. All doses used were selected based on the results obtained from preliminary experiments. The leaf dip assay method [8] was employed using Chinese cabbage (*Brassica campestris* L. ssp. *Pekinensis*) leaves approximately 7 cm in diameter, which were dipped in either co-solvent only (acetone) control or insecticide solutions, then allowed to air dry for 2 h prior to use [8]. For each treatment, 30 newly molted fourth-instar larvae were placed on each treated leaf. Assays were conducted in sterile glass Petri dishes (9 cm in diameter) at 25 ± 2 °C, 70 ± 5% relative humidity with a photoperiod of 16 h light/8 h dark. After 24 h, midguts and fat bodies of surviving larvae were isolated and flash-frozen in liquid nitrogen, and stored at −80 °C prior to use. Three independent biological replicates were performed for all treatments.

### 4.7. Effect of PBO on Toxicity of Insecticides

PBO was used as the synergist in this study. For each insecticide, five different concentrations of insecticide solutions were prepared and tested as mentioned above. Insecticide toxicity in the presence or absence of PBO was assessed using the same bioassay method described above. Thirty fourth instar *S. exigua* larvae were tested for each concentration of an insecticide. PBO (25 mg/mL) were prepared in acetone. After applying PBO (10 μg/larvae) to the dorsal prothorax of individual larvae for 1 h, the larvae were placed in sterilized Petri dishes containing fresh Chinese cabbage leaf discs with different concentrations of insecticides [3]. Mortality was recorded after 48 h and the LC_50_ values were calculated [3]. The synergism ratio (SR) was calculated by dividing the LC_50_ of insecticide alone by LC_50_ of insecticide + PBO [3]. Each experiment was performed in triplicate.

### 4.8. dsRNA Synthesis

For dsRNA synthesis, a 405 bp fragment from *CYP9A105* and a 688 bp fragment from green fluorescent protein (GFP) (GenBank accession ACY56286) were first amplified by PCR. The primers used for the *CYP9A105* and GFP amplifications were designed to add the T7 polymerase promoter sequence at the 5′ ends. Two pairs of primers (CYP9A105-F and T7CYP9A105-R, T7CYP9A105-F and CYP9A105-R) were used to amplify *CYP9A105* (Table 2). As a control, dsGFP was synthesized using the same method by two pairs of primers (GFP-F and T7GFP-R, T7GFP-F and GFP-R) (Table 2). dsCYP9A105 and dsGFP were prepared from the purified PCR-generated templates according to the instructions provided with the T7 RiboMax Express RNAi System kit (Promega, Madison, WI, USA). The resulting dsRNAs were quantified by UV-Vis spectroscopy and analyzed by agarose gel electrophoresis, and then stored at −80 °C prior to use.

### 4.9. RNA Interference Bioassays

For RNAi bioassays, double-stranded RNAs (dsRNA) dissolved in diethylpyrocarbonate (DEPC)-treated water were injected into fourth instar (day 1) larvae of *S. exigua* using a manual microinjector (model no. MS05, Chengdu Centome Company Ltd., Chengdu, China) as previously described [3,8], using 2 μL (3.0 μg) aliquots of dsRNA injected into larvae between the second and the third abdominal segments, and larvae injected with dsGFP served as control. Midguts and fat bodies of surviving larvae (four larvae) were collected from *S. exigua* after injection with either dsCYP9A105 or dsGFP for 24 h, 48 h and 72 h, respectively. All experiments were performed in triplicate (three biological replicates). For each replicate, midguts from four surviving larvae of *S. exigua* were collected for total RNA extraction. The RNA extraction and quantitative RT-PCR procedures used were described above.

To evaluate the role of *CYP9A105* in the detoxification of pyrethroids, fourth instar (day 1) larvae were injected with dsCYP9A105 or dsGFP. After dsRNA delivery, 30 larvae per treatment were exposed to deltamethrin, *α*-cypermethrin, fenvalerate or control treatments applied to Chinese cabbage leaves using the leaf dip method at LC_50_ dosage, as described above. After 48 h, mortality rates were recorded for each treatment from bioassays performed in triplicate.

### 4.10. Statistical Analysis

All data were analyzed using the SPSS 13.0 Software Package (SPSS Inc., Chicago, IL, USA). One-way ANOVA followed by the Duncan’s multiple range test was employed to analyze differences among different tissues and development stages. The Student’s *t*-test was used to analyze data from the RNAi and feeding experiments with different pyrethroids. Statistical differences were considered as significant at *p* < 0.05.

## Figures and Tables

**Table 1:** Effect of PBO on the toxicity of pyrethroids to fourth instar *S. exigua* larvae.

Treatment	Slope ± SE	*r*	*χ*^2^	LC_50_ (mg/L) (95% CL)	df	SR
α-cypermethrin	1.791 ± 0.038	0.9856	1.964	0.266 (0.224–0.315)	4	-
α-cypermethrin + PBO	1.817 ± 0.075	0.9549	4.757	0.057 (0.041–0.081)	4	4.63
deltamethrin	1.866 ± 0.039	0.9915	1.140	0.431 (0.362–0.514)	4	-
deltamethrin + PBO	1.805 ± 0.053	0.9881	1.216	0.114 (0.090–0.145)	4	3.78
fenvalerate	1.693 ± 0.038	0.9817	2.430	10.915 (9.190–12.965)	4	-
fenvalerate + PBO	1.676 ± 0.082	0.9316	4.454	2.120 (1.463–3.071)	4	5.15

CL: confidence limits. df: degrees of freedom. SR (synergism ratio) = LC_50_ of insecticide alone/LC_50_ of insecticide + PBO. *r*: correlation coefficient. *χ*^2^: Chi-square value.

**Table 2:** Primers used in this study.

Function	Primer Name	Primer Sequence (5′-3′)
Full-length	CYP9A105FullF	ATGATTATCTTTTTCATTTGGTTG
	CYP9A105FullR	TTATTTTCTCAGTCGGAACCTAAG
Real-time PCR		
CYP9A105	CYP9A105F1	TCCACCACGATCCTCAGTAC
	CYP9A105R1	TCATCTCGCAAAGAGCAAAT
β-actin	β-actinF	TGCGTGACATCAAGGAGAAGC
	β-actinR	CCATACCCAGGAAGGAAGGCT
dsRNA synthesis		
dsCYP105	CYP9A105-F	TGTTCTTTGTGGCTGGTTTTG
	T7CYP9A105-R	aatacgactcactataggTCTTGTGTTTATTTTCGTCGG
	T7CYP9A105-F	aatacgactcactataggTGTTCTTTGTGGCTGGTTTTG
	CYP9A105-R	TCTTGTGTTTATTTTCGTCGG
dsGFP	GFP-F	AAGGGCGAGGAGCTGTTCACCG
	T7GFP-R	aatacgactcactataggCAGCAGGACCATGTGATCGCGC
	T7GFP-F	aatacgactcactataggAAGGGCGAGGAGCTGTTCACCG
	GFP-R	CAGCAGGACCATGTGATCGCGC
